# Comparative biosafety and efficacy of *Pseudomonas fluorescens* PFS and *Lactiplantibacillus plantarum* ZPZ against *Ralstonia solanacearum*

**DOI:** 10.1038/s41598-025-26624-7

**Published:** 2025-11-20

**Authors:** Astghik Pepoyan, Michael Leonidas Chikindas

**Affiliations:** 1 Department of Food Safety and Biotechnology, Armenian National Agrarian University, 74 Teryan St., 0009 Yerevan, Armenia; 2 Health Promoting Naturals Laboratory, School of Environmental and Biological Sciences, Rutgers State University, 65 Dudley Rd., New Brunswick, NJ 08901 USA; 3 Department of General Hygiene, I.M. Sechenov First Moscow State Medical University, Trubetskaya Str. 8, Bldg. 2, Moscow, Russia 119991

**Keywords:** *Lactiplantibacillus plantarum*, *Pseudomonas fluorescens*, *Ralstonia solanacearum*, Biocontrol, Biosafety, One Health, Microbial technology, Biotechnology, Microbiology, Plant sciences

## Abstract

In light of increasing environmental variability, the biocontrol of *Ralstonia solanacearum*, one of the most destructive plant pathogens worldwide, remains a pressing challenge. While *Pseudomonas fluorescens* strains have demonstrated biocontrol efficacy, concerns remain regarding their ecological safety within the One Health framework. These include the production of antimicrobial compounds contributing to resistance gene dissemination, variable efficacy across environments and strains, and occasional adverse effects on plant immunity. As a potential alternative, *Lactiplantibacillus plantarum* ZPZ, a lactic acid bacterium with GRAS status and cross-domain probiotic features, was comparatively assessed for its efficacy and biosafety profile against *R. solanacearum*. In vitro experiments showed that the cell-free supernatant of *Lpb. plantarum* ZPZ inhibited the growth of *R. solanacearum* by 72.46 ± 14.42% based on OD_600_ measurements (*P* < 0.05). Compared to *P. fluorescens* spp., which require favorable conditions and may carry risks for environmental or human health, *Lpb. plantarum* ZPZ exhibited a metabolically versatile and biosafe profile suitable for sustainable biocontrol strategies. These findings underscore the importance of weighing both efficacy and ecological safety when selecting microbial agents for plant disease management. *Lpb. plantarum* ZPZ emerges as a promising candidate for environmentally responsible control of bacterial wilt.

**Supplementary Information:**

The online version contains supplementary material available at 10.1038/s41598-025-26624-7.

## Introduction

*Ralstonia solanacearum*, a Gram-negative, xylem-invading phytopathogen^1^, is widely recognized as the causative agent of bacterial wilt, a disease responsible for significant yield losses in economically important solanaceous crops such as tomato and potato^2^. Reported yield reductions vary widely, ranging from 20% to complete crop failure (100%), depending on crop type and environmental conditions^1^. The pathogen’s high adaptability, persistence in diverse environments (including soil and water), broad host range, and genetic diversity contribute to its notoriety as one of the most intractable bacterial threats in modern agriculture^3^.

Previous research has demonstrated that biological control agents (BCAs) can effectively suppress *R. solanacearum*. Meta-analyses indicate significant reductions in disease incidence and severity, as well as increased plant growth and yield, particularly with *Pseudomonas* spp. compared to *Bacillus* spp.^2^. Various BCAs, including *Bacillus velezensis*, *Trichoderma* spp., and *Pseudomonas fluorescens*, exert their effects through antimicrobial compound production, competition, and activation of host defenses^3^. Specifically, *P. fluorescens* enhances plant resistance by inducing defense-related enzymes^4^. Its volatile organic compounds inhibit *R. solanacearum* growth and virulence by targeting essential metabolic pathways^5^. Furthermore, synergistic application of *P. fluorescens* with plant extracts, such as *Rosmarinus officinalis*, has shown enhanced disease suppression under greenhouse conditions^6^.

Microbial solutions, as key elements of green technologies, support sustainable practices by mitigating environmental degradation and enhancing ecological resilience^7,8^. A notable example is the use of probiotics—microbes known for their health-promoting properties in humans^9–11^. Initially associated with human health, probiotics are now applied in plant production systems, improving host immunity, growth performance, and tolerance to biotic and abiotic stressors^12,13^.

*Pseudomonas* encompasses both pathogenic and commensal strains. Certain species are widely integrated into sustainable agricultural strategies due to their biocontrol efficacy and plant growth–promoting traits. Members of this genus, particularly *P. fluorescens*^14^ and *P. putida*^15^, are well-characterized plant growth–promoting rhizobacteria (PGPR), renowned for antimicrobial activity, nutrient solubilization, and induction of systemic resistance in plants.

Emerging evidence shows that some *Pseudomonas* spp., while beneficial under certain conditions, may display undesirable or pathogenic traits depending on the ecological context. For example, *P. fluorescens* strain P97-38 enhances *Arabidopsis thaliana* resistance to *P. syringae* DC3000 but concurrently increases susceptibility to the herbivore *Spodoptera littoralis*. This trade-off is linked to hormonal signaling: salicylic acid-mediated defenses suppress jasmonic acid-dependent responses required for herbivore resistance^16^. Such examples highlight the complexity and occasionally conflicting outcomes of employing beneficial microbes in plant immunity.

Certain *P. fluorescens* strains, such as CFBP2392, produce antifungal antibiotics against pathogens like *Rhizoctonia solani*^16,17^. While antibiotic production is generally advantageous for biocontrol, it may entail risks, including potential emergence of antibiotic resistance, which remains insufficiently assessed. The use of *Pseudomonas* spp. as biocontrol agents is thus not without risks. Their diverse secondary metabolites, while targeting pathogens, may also exert unintended non-target effects, contributing to biosafety concerns and potential human health implications^18,19^.

These findings underscore the necessity of carefully evaluating both benefits and potential risks when utilizing microbial agents, particularly *Pseudomonas* spp., in agroecosystems. Considering these factors and ongoing environmental changes, it is hypothesized that from a biosafety perspective, comparative assessment of “safe bacteria” and those classified as “potentially problematic” or “context-dependent” is beneficial^16,17^. In this context, *Lactiplantibacillus plantarum* ZPZ^20,21^, a lactic acid bacterium with GRAS status, is proposed as a biosafe and ecologically adaptive alternative to *Pseudomonas* spp. This is particularly relevant under shifting climatic conditions where antibiotic resistance, horizontal gene transfer, and immune system trade-offs may compromise the reliability of *Pseudomonas*-based strategies. Such comparative evaluations may contribute to more consistent and ecologically sound biocontrol approaches.

*Lpb. plantarum* ZPZ has demonstrated broad-spectrum probiotic properties in both medical and agricultural contexts^20–22^. Its cell-free supernatants (CFS) exhibited strong inhibitory activity against multidrug-resistant *Klebsiella pneumoniae* isolates from clinical, veterinary, and aquatic sources. With a favorable safety profile, characterized by the absence of pathogenicity and antibiotic biosynthesis^21,22^ (Supplementary Tables S1 and S2), *Lpb. plantarum* ZPZ is a promising candidate for environmentally friendly biocontrol applications.

This study evaluates the biocontrol potential of *P. fluorescens* PFS and *Lpb. plantarum* ZPZ against *R. solanacearum* isolated from tomato plants exhibiting bacterial wilt symptoms. It is hypothesized that *Lpb. plantarum* ZPZ, a LAB with GRAS status and previously demonstrated cross-domain probiotic activity, may serve as a safer and more ecologically sustainable alternative for the integrated management of bacterial wilt in agricultural ecosystems.

## Materials and methods

### Study design

This study combined a literature-based analysis with preliminary microbiological experiments to evaluate the environmental and probiotic potential of *Lactiplantibacillus plantarum* ZPZ, focusing on its antagonistic activity against *Ralstonia solanacearum* isolated from tomato plants affected by bacterial wilt. The theoretical component was based on a review of relevant literature, emphasizing the antagonistic mechanisms of *Pseudomonas* spp., particularly *P. fluorescens* PFS in phytopathogen control.

### Bacterial strains and culture conditions

The *Lpb. plantarum* ZPZ strain was kindly provided by the International Association for Human and Animals Health Improvement (Armenia), and the *R. solanacearum* strain was obtained from the culture collection of the Armenian National Agrarian University (Armenia). Fresh cultures of *Lpb. plantarum* ZPZ were grown in de Man, Rogosa, and Sharpe (MRS) broth (Fisher Scientific, Pittsburgh, PA, USA) at 37 °C for 48 h. *R. solanacearum* (formerly classified as *Pseudomonas solanacearum* and *Burkholderia solanacearum*) was cultured on a selective medium containing bacteriological agar (17.0 g; Merck Life Science LLC, Moscow, RF), casein peptone (11.0 g; Merck Life Science LLC, Moscow, RF), and d-(+)-glucose (5.0 g; Merck Life Science LLC, Moscow, RF), following the protocol described by Atlas^23^.

### Preparation of cell-free supernatant (CFS)

After incubation, the *Lpb. plantarum* ZPZ culture was adjusted to 1.5 × 10^8^ CFU/mL, centrifuged at 5000×*g* for 15 min at 4 °C, and the supernatant was collected and filtered through a 0.22 μm pore-size membrane filter (Millex-GV, Sigma-Aldrich, St. Louis, MO, USA) to obtain sterile CFS.

### Liquid culture antagonism assay

For the control group, 0.1 mL of *R. solanacearum* (1.5 × 10^8^ CFU/mL) was added to 9.9 mL of medium without agar. For the experimental group, 0.1 mL of CFS and 0.1 mL of *R. solanacearum* suspension were added to 9.8 mL of the same medium. Both media were adjusted to the same initial pH. Cultures were incubated at 28 °C for 48 h, and the inhibitory effect of the CFS was determined by measuring optical density at 600 nm (OD_600_) and viable cell counts. Antimicrobial activity was calculated using the formula:

$${\text{Inhibition }}\left( \% \right) = \left[ {1 - \left( {{\text{Titer}}/{\text{OD}}_{600} \left( {\text{treated sample}} \right)/{\text{Titer}}/{\text{OD}}_{600} \left( {{\text{control}}} \right)} \right)} \right] \times 100.$$

### Microplate inhibition assay

Inhibition assays were also performed in 96-well microplates. Fifty:fifty mixtures of CFS and Miller Luria–Bertani (LB) broth (HiMedia Laboratories, Pennsylvania, USA) were prepared for experimental wells. As a negative control, *R. solanacearum* was grown in 50:50 mixtures of LB and sterile water. Each well was inoculated with 2 µL of *R. solanacearum* suspension. Plates were incubated at 28 °C for 3 days, and bacterial growth was monitored by OD_600_ measurements at 24 h and 72 h.

Microplate assay results are presented in detail in Supplementary Table S3.

### Molecular confirmation of *R. solanacearum*

The identity and purity of the *R. solanacearum* culture were confirmed by PCR using primers Ps-1 and Ps-2, as recommended by the European Commission^24^.

### Comparative analysis with *Pseudomonas fluorescens*

The biocontrol potential of *Lpb. plantarum* ZPZ was compared with published data on *P. fluorescens*, especially regarding its effectiveness against *R. solanacearum*. Although direct comparisons between in vitro results and field trials are limited due to differing conditions, such comparisons can provide preliminary insights into the relative efficacy of microbial strains.

### Statistical analysis

All experiments were performed in triplicate (n = 3). Results are presented as mean ± standard deviation (SD). Data normality was tested using the Shapiro–Wilk test. Statistical significance was assessed with two-tailed Student’s t-tests (Microsoft Excel 2016), with *P* < 0.05 considered significant.

## Results

### Literature-based insights into *Pseudomonas fluorescens* spp. for *Ralstonia solanacearum* control

Previous studies have demonstrated that several *P․ fluorescens* spp. offer promising biocontrol potential against *R. solanacearum* through diverse mechanisms (Table 1). For instance, *P. fluorescens* PFS strains achieved up to 96% disease suppression in tomato field trials^25^, while *P. fluorescens* VSMKU3054 induced systemic resistance by enhancing key plant defense enzymes, including peroxidase (POX), polyphenol oxidase (PPO), lipoxygenase (LOX), and phenylalanine ammonia lyase (PAL)^4^. However, such approaches often depend on successful root colonization and are sensitive to environmental conditions and timing of application. Other strategies have explored the role of volatile organic compounds (VOCs) produced by *P. fluorescens* WR-1, which showed inhibitory effects on virulence-related proteins of *R. solanacearum*, although their efficacy remains concentration-dependent and lacks field-level validation^5^. Moderate inhibition levels were reported for *P. fluorescens* sp., reducing disease severity index (DSI) by 65% and producing a 2.2 cm inhibition zone^6^. Notably, synergistic approaches combining *P. fluorescens* with plant-derived compounds, such as *Rosmarinus officinalis* extract, led to enhanced biocontrol efficacy, although the individual contribution of each component remains unclear (Table 1).

**Table 1:** Summary of *Pseudomonas* control approaches applied against *Ralstonia solanacearum.*

Agent/Method	Efficacy	Limitations	References
*P. fluorescens* VSMKU3054	Induced high levels of peroxidase, polyphenol oxidase, lipoxygenase, phenylalanine ammonia lyase, and phenol; enhanced defense responses in tomato; suppressed *R. solanacearum* wilt	Requires prior root colonization; enzyme induction varies depending on the time after inoculation	^4^
*P. fluorescens* PFS	22.33 mm zone of inhibition against *Ralstonia* spp. in vitro; up to 96% disease suppression in tomato field trials	Requires optimal environmental conditions; strain- and environment-dependent	^25^
*P. fluorescens* WR-1 (via volatile organic compounds (VOCs))	VOCs inhibit growth and virulence traits; affect antioxidant, metabolism, and virulence-related proteins	VOCs have concentration-dependent efficacy; actual field performance has not been fully assessed	^5^
*P. fluorescens* isolate	65% reduction in disease severity index; inhibition zone of 2.2 cm; decreased bacterial population in plant tissues; enhanced resistance induction	Lower efficacy compared to chemical or plant extract treatments; may require formulation support	^6^
*R. officinalis* extract + *P. fluorescens* mixture	77.9% reduction in DSI; 50% pathogen reduction in stem; significantly increased plant growth (fresh weight: 169.9 g; dry weight: 170.4 g); synergistic action	The inhibition zone was not independently reported; stability and compatibility of the formulation may vary	^6^

### In vitro antagonistic activity of *Lactiplantibacillus plantarum* ZPZ cell-free supernatant

As shown in Table 2, treatment with the CFS of *Lpb. plantarum* ZPZ significantly inhibited *R. solanacearum* growth, reducing OD_600_ by 72.46 ± 14.42% and viable cell counts by 88.89 ± 45.26% compared to the untreated control (*P* < 0.05). The high variability in viable counts likely reflects technical limitations of the plating method rather than biological differences. Overall, both assays confirm the antimicrobial activity of ZPZ, with OD_600_ measurements providing a more consistent and robust estimate of growth inhibition. In addition, similar inhibitory effects were observed in microplate assays using 50:50 mixtures of *Lpb. plantarum* ZPZ CFS and LB broth, confirming the antimicrobial activity observed in the standard liquid culture experiments (Supplementary Table S3).

**Table 2:** In vitro antagonistic effect of cell-free supernatant of *Lactiplantibacillus plantarum* ZPZ against *Ralstonia solanacearum*, based on OD_600_ and viable counts (CFU/mL).

Sample	Final OD_600_ (mean ± SD)	Final viable count—liquid culture (CFU/mL, mean ± SD)	Growth inhibition OD_600_ (%)	Growth inhibition CFU (%)
Control	1.543 ± 0.059	1.50 × 10^8^ ± 0.30 × 10^8^	–	–
With CFS	0.425 ± 0.083	1.67 × 10^7^ ± 7.80 × 10^6^	72.46 ± 14.42	88.89 ± 45.26*

Data are shown as mean ± SD (n = 3). Normality was verified via Shapiro–Wilk test. Statistical significance was assessed using two-tailed Student’s t-tests (*P* < 0.05). Microplate assay results showed similar inhibitory trends as liquid culture experiments (Supplementary Table S2). *The high variability in viable counts likely reflects technical limitations of the plating method rather than biological differences

## Discussion

This study presents a comparative assessment of two microbial candidates for the biocontrol of *R. solanacearum*: *P. fluorescens* PFS and the One Health probiotic *Lpb. plantarum* ZPZ. While *P. fluorescens* strains such as PFS have demonstrated high efficacy (up to 96%) in field trials^25^, their application is often limited by strain specificity, environmental sensitivity, and their capacity to produce antibiotic compounds^4,5^, which raises biosafety concerns in the context of resistance gene dissemination^26^.

While Raman et al. highlighted the limited in vitro frameworks, the pH- and temperature-dependent activity, and the absence of integrated mechanistic analyses for LAB as plant probiotics, the present study focuses on prioritizing functional efficacy within a One Health paradigm when selecting biocontrol agents. This study advocates continued broad‑scale LAB screening against bacterial wilt, grounded in their diverse genetic potentials, while directly comparing field‑validated *P. fluorescens* PFS with *Lpb. plantarum* ZPZ based on genomic determinants of organic acid biosynthesis, bacteriocin‑like peptide production, and folate/cofactor pathways (Table 3).

**Table 3:** Comparative features of *Pseudomonas fluorescens* PFS and *Lactiplantibacillus plantarum* ZPZ.

Feature	*P. fluorescens* PFS	*Lpb. plantarum* ZPZ	References
In vitro antagonistic activity	22.33 mm inhibition zone; reduction of *R. solanacearum* wilt by up to 96% in tomato field trials^25^	72.46% OD_600_ inhibition (Table 2)	This study
Mechanism(s) of action	Antibiotic and lytic enzyme production Induction of plant defense enzymes (POX, PPO, LOX, PAL) VOC-mediated inhibition	Organic acids and bacteriocin-like compounds in CFS Biofilm formation and competitive exclusion Folate-mediated redox modulation	^22,4,5,31^
Environmental sensitivity	Efficacy is strongly strain- and environment-dependent; requires optimized timing and conditions for root colonization	Broadly effective in vitro; genomic features (vitamin/cofactor pathways) suggest resilience to varying ecological contexts	^5,6,28^
Field-level validation	Demonstrated up to 96% disease suppression in multiple tomato trials	Not yet tested in planta; in vitro efficacy established, but field trials remain to be performed	^25^
Safety profile	Potential risk of antibiotic-resistance gene dissemination; some strains produce broad-spectrum antibiotics	GRAS status; no known antibiotic-biosynthesis genes; absence of pathogenicity	^17,22,26^
Vitamin/cofactor biosynthesis genes	Limited genomic capacity for vitamin synthesis beyond basic cofactors	> 110 genes for biosynthesis of folate (B9), riboflavin (B2), pyridoxine (B6), biotin (B8), thiamin (B1); plus 5-FCL–like, heme/siroheme and molybdenum cofactor pathways	^22,34^
Multimodal potential	Primarily direct antagonism and ISR induction	Combines direct antagonism, nutrient supplementation (via vitamins), redox balancing, and biofilm-mediated colonization	^28,33,35^

According to current investigations, CFS of *Lpb. plantarum* ZPZ demonstrated substantial in vitro antagonism against *R. solanacearum*, with OD_600_ measurements indicating a 72.46 ± 14.42% growth inhibition (Table 2). Its previously documented antagonism against multidrug-resistant *Klebsiella pneumoniae*^21^ supports its cross-domain relevance, particularly under One Health frameworks^28^. Our findings align with earlier studies reporting LAB-mediated antagonism against *R. solanacearum*^29,30^. Together, these studies imply that LAB strain ZPZ’s efficacy is part of a broader pattern of LAB being effective biocontrol agents. These features are increasingly valuable under shifting ecological and climate conditions, where robust, biosafe biocontrol agents are urgently needed. Among the noteworthy features of *Lpb. plantarum* ZPZ is its ability to biosynthesize folate (vitamin B9)^22^, a metabolite that indirectly enhances plant health by contributing to redox homeostasis, nucleotide synthesis, and the activation of defense-related pathways^31,32^. Exogenous folic acid application has also been shown to enhance chlorophyll synthesis and overall growth in common bean (*Phaseolus vulgaris* L.), increasing the Dark Green Color Index, leaf area, and biomass compared to untreated plants^33^. Furthermore, folate-dependent one-carbon metabolism modulates plant immunity: disruption of the folate pathway primes a stronger defense response in *Arabidopsis thaliana*, leading to enhanced resistance against *Pseudomonas syringae* DC3000 via activation of a methionine synthase-mediated immune state^34^.

Previously, whole-genome analysis revealed that *Lpb. plantarum* ZPZ contains over 110 genes involved in vitamin and cofactor biosynthesis, including complete sets for folate, riboflavin, pyridoxine, biotin, and thiamin synthesis. It also harbors genes encoding for the 5-formyltetrahydrofolate cyclo-ligase-like protein and components of heme and siroheme biosynthetic pathways. These genomic features suggest a broader metabolic potential compared to other lactobacilli, supporting its capacity to influence plant physiological and immune functions beyond direct antagonism^22^. Notably, the genome also includes genes associated with the biosynthesis of the molybdenum cofactor, an essential enzyme cofactor known to influence redox reactions and cellular homeostasis in both microbial and plant systems. The synergistic effect of folate production with other traits, such as organic acid secretion^22^, biofilm formation, competitive exclusion, and redox modulation, suggests a multimodal mechanism of action for ZPZ^35,36^, which may render it more adaptable across diverse ecological conditions. This genome-enabled functionality provides a strong foundation for future exploration of this strain in sustainable biocontrol applications, either alone or in consortia with complementary microbes like *P. fluorescens*.

Thus, while *P․ fluorescens* strains may provide more effective disease suppression under optimal field conditions, they simultaneously raise serious concerns from a One Health perspective. In particular, the antibiotic-producing activity of *P. fluorescens* can contribute to emerging problems, given that the spread of antibiotic resistance remains one of the central challenges for human, animal, and environmental health^37^. In contrast, *Lactiplantibacillus plantarum* ZPZ represents a safer, metabolically versatile, and ecologically sustainable alternative for the integrated management of bacterial wilt.

## Conclusion

This study provides a comparative evaluation of *P. fluorescens* PFS and *Lpb. plantarum* ZPZ in managing *R. solanacearum*, a major phytopathogen in global agriculture. While *P. fluorescens* demonstrated superior suppression efficacy under optimal conditions, its antibiotic-producing nature and strain-specific limitations raise biosafety and ecological concerns. In contrast, *Lpb. plantarum* ZPZ offered a moderate but significant inhibitory effect, reducing *R. solanacearum* growth by 72.46 ± 14.42%, and presents multiple advantages: GRAS status, vitamin biosynthesis capacity, lack of pathogenic traits, and previously confirmed activity against multidrug-resistant *K. pneumoniae*.

This study presents some preliminary in vitro data. However, its limitations, primarily due to the preliminary nature of the study, should be acknowledged. These include the lack of validation in biologically relevant systems (plants, soil, field) and the lack of direct comparative data with established alternatives. Further experimental assessment of ecological safety beyond the general taxonomy should also be a goal of future research. In addition, mechanistic and practical application insights are essential for validation of the proposed approach. These gaps need to be addressed before *Lpb. plantarum* ZPZ can be confidently promoted as a viable and environmentally responsible biocontrol agent for bacterial wilt. Future studies should aim to validate its efficacy under field conditions and assess its interaction with plant immune systems in complex soil microbiomes. Nevertheless, given its biosafety profile, metabolic flexibility, and potential for application across sectors, *Lpb. plantarum* ZPZ represents a promising, and hopefully, environmentally aligned candidate for biocontrol strategies.

## Supplementary Information

Below is the link to the electronic supplementary material.

Supplementary Material 1

## Data Availability

All data generated or analyzed during this study are included in this published article.
