# Supplementary material for: Comparative biosafety and efficacy of Pseudomonas fluorescens PFS and Lactiplantibacillus plantarum ZPZ against Ralstonia solanacearum
Source: Sci Rep. 2025 Nov 20;15:38443. doi: 10.1038/s41598-025-26624-7 (PMC12630949; doi:10.1038/s41598-025-26624-7)
Supplement: Supplementary file 1 — Supplementary Material 1 [file 41598_2025_26624_MOESM1_ESM.docx]

**Table S1.** Genomic features of *Lactiplantibacillus plantarum* ZPZ related to antimicrobial activity and safety profile*

| **Category** | **Example CDS/functions** | **Functional implication** |
| --- | --- | --- |
| **Organic acid metabolism** | Lactate dehydrogenase; 1,3-propanediol dehydrogenase | Acidification of growth medium; inhibition of competing bacteria |
| **Lipid & glycolipid metabolism** | 1,2-diacylglycerol 3-glucosyltransferase | Production of glycolipids that destabilize microbial membranes |
| **Isoprenoid/fatty acid metabolism** | Farnesyl diphosphate synthase | Formation of terpenoid intermediates with antimicrobial potential |
| **Oxidative stress / oxidoreductases** | Multiple oxidoreductases, dehydrogenases | Generation of hydrogen peroxide (H₂O₂) and other reactive metabolites inhibitory to pathogens |
| **Small peptide CDSs (<200 aa)** | Hypothetical proteins with peptide-like features | Possible bacteriocin-like activity (non-classical bacteriocins) |
| **Absence of antibiotic biosynthetic clusters** | No canonical secondary metabolite clusters detected | Confirms that inhibitory activity is not due to classical antibiotics |
| **Absence of virulence determinants** | No hemolysins, toxins, or type III secretion system genes identified | Indicates lack of pathogenicity |
| **Absence of resistance genes** | No acquired antibiotic resistance genes annotated | Supports ecological and medical safety |
| *The genome of *Lactiplantibacillus plantarum* ZPZ was previously sequenced and deposited in the DDBJ/EMBL/GenBank database (accession no. VRTR00000000)^22^. Genome annotation was performed using the RAST server, which identified 3402 coding sequences and 71 RNA genes organized into 248 functional subsystems. | | |

**Table S2.** Functional gene clusters* of *Lactiplantibacillus plantarum* ZPZ relevant to antimicrobial activity, biofilm formation, and plant health

| **Category** | **Example genes** | **Potential functional role** |
| --- | --- | --- |
| **Organic acid metabolism** | Pyruvate oxidase, Lactate dehydrogenase, 1,3-propanediol dehydrogenase | Production of lactic and acetic acids, acidification of medium, inhibition of pathogen growth |
| **Oxidative stress & reactive compounds** | Flavodoxin, Hydrogen peroxide-inducible genes activator, Multiple oxidoreductases | Generation of H₂O₂ and reactive oxygen species, creating antimicrobial stress for pathogens |
| **Membrane-active molecules** | 1,2-diacylglycerol 3-glucosyltransferase, glycolipid synthesis genes | Production of glycolipids and exopolysaccharides that destabilize pathogen membranes and contribute to biofilm structure |
| **Small peptide coding sequences (<200 aa)** | Hypothetical peptides with bacteriocin-like features | Possible non-classical bacteriocins contributing to direct inhibition of pathogens |
| **Vitamin & folate biosynthesis** | Folate biosynthesis enzymes, riboflavin and thiamine pathway genes | Contribution to plant growth promotion, host nutritional support, and stress tolerance |
| **Stress response regulators** | LysR-family regulators, glutaredoxins | Regulation of redox balance, stress adaptation, potential modulation of plant–microbe interactions |
| *The genome of *Lactiplantibacillus plantarum* ZPZ was previously sequenced and deposited in the DDBJ/EMBL/GenBank database (accession no. VRTR00000000)^22^. Genome annotation was performed using the RAST server, which identified 3402 coding sequences and 71 RNA genes organized into 248 functional subsystems. | | |

**Table S3.** Inhibitory effect of the cell free supernatant from *Lactiplantibacillus plantarum* ZPZ against *Ralstonia solanacearum:* microplate assay

| **Hour** | **Control (OD_600_)** | **Treated (OD_600_)** | **Inhibition (%)** |
| --- | --- | --- | --- |
| 24h | 0.67 ± 0.072 | 0.306 ± 0.077 | 54.33 ± 10.32 |
| 72h | 1.1 ± 0.043 | 0.267 ± 0.123 | 75.77 ± 11.097 |
